# Oncofertility: combination of ovarian stimulation with subsequent ovarian tissue extraction on the day of oocyte retrieval

**DOI:** 10.1186/1477-7827-11-19

**Published:** 2013-03-05

**Authors:** Ralf Dittrich, Laura Lotz, Andreas Mueller, Inge Hoffmann, David L Wachter, Kerstin U Amann, Matthias W Beckmann, Thomas Hildebrandt

**Affiliations:** 1 Department of Obstetrics and Gynecology, Erlangen University Hospital, Universitätsstrasse 21-23, 91054, Erlangen, Germany; 2 Department of Obstetrics and Gynecology, Karlsruhe Hospital, Moltkestrasse 90, 76133, Karlsruhe, Germany; 3 Department of Pathology, Erlangen University Hospital, Krankenhausstrasse 8-10, 91054, Erlangen, Germany

**Keywords:** Fertility preservation, Ovarian tissue, Ovarian stimulation, IVF, Oocyte cryopreservation, Ovarian tissue cryopreservation

## Abstract

**Background:**

New anticancer treatments have increased survival rates for cancer patients, but often at the cost of sterility. Several strategies are currently available for preserving fertility. However, the chances of achieving a pregnancy with one technique are still limited. A combination of methods is therefore recommended in order to maximize women’s chances of future fertility. In this retrospective study, ovarian stimulation with subsequent ovarian tissue extraction on the day of oocyte retrieval were combined and the quality of the ovarian tissue, the numbers and quality of oocytes, time requirements, and the safety of the strategy were examined.

**Methods:**

Fourteen female patients suffering from malignant diseases underwent one in vitro fertilization cycle. Different stimulation protocols were used, depending on the menstrual cycle. Transvaginal oocyte retrieval was scheduled 34–36 h after human chorionic gonadotropin administration. Immediately afterwards, ovarian tissue was extracted laparoscopically.

**Results:**

A mean of 10 oocytes were retrieved per patient, and 67% of the oocytes were successfully fertilized using intracytoplasmic sperm injection. No periprocedural complications and no complications leading to postponement of the start of chemotherapy occurred. The ovarian tissues were of good quality, with a normal age-related follicular distribution and without carcinoma cell invasion.

**Conclusions:**

An approach using ovarian stimulation first, followed by laparoscopic collection of ovarian tissue, is a useful strategy for increasing the efficacy of fertility preservation techniques. The ovarian tissue is not affected by prior ovarian stimulation.

## Background

In recent years, progress in the diagnosis and treatment of oncological diseases has led to considerable improvements in the survival prognosis, particularly in children and adolescent cancer patients. Unfortunately, aggressive chemotherapy and radiotherapy often cause infertility due to massive destruction of the ovarian reserve, resulting in premature ovarian failure. These women have to face years of hormone replacement therapy and the prospect of infertility, which causes psychological stress [1,2].

Several strategies are currently available for preserving fertility, depending on the risks and probability of gonadal failure, the patient’s general health at diagnosis, and the partner’s status. These strategies include transposition of the ovaries before radiotherapy, ovarian stimulation followed by cryopreservation of fertilized oocytes or unfertilized oocytes, cryopreservation of in vitro–matured oocytes, cryopreservation and transplantation of ovarian tissue, and administration of gonadotropin-releasing hormone (GnRH) agonists [3,4].

Although the variety of fertility preservation strategies available enables cancer patients to have children using their own gametes after overcoming their disease, most of these techniques are still experimental and the efficacy of the individual techniques is limited. A combination of methods is therefore recommended in order to maximize women’s chances of future fertility [5].

In this study, ovarian stimulation and ovarian tissue cryopreservation were combined as a strategy for fertility preservation in cancer patients. The aim was to evaluate whether ovarian stimulation affects the quality of ovarian tissue. The numbers and quality of oocytes, time requirements, and the safety of this strategy were also examined.

## Methods

Fourteen patients between 24 and 35 years of age (median 29) were included in the retrospective study. They were all suffering from malignant diseases (Table 1) and wanted to preserve their fertility for a future pregnancy. None of the patients had been treated with chemotherapy or radiotherapy before the fertility preservation procedure. All patients provided informed consent for ovarian stimulation and ovarian tissue cryopreservation, after receiving counseling on alternative options for fertility preservation techniques. Three patients also wished to be treated with GnRH agonists during chemotherapy.

**Table 1:** Main characteristics and stimulation outcome in patients who provided informed consent for ovarian stimulation and ovarian tissue cryopreservation

**Patient**	**Cancer type**	**Age**	**Days of stimulation**	**Aspirated oocytes (n)**	**Cryopreserved zygotes after ICSI (n)**
1	Acute myelogenous leukemia	28	9	5	5
2	Hodgkin’s lymphoma	25	6	7	4
3	Cervix carcinoma	31	10	10	5
4	Leiomyosarcoma	26	11	13	11
5	Breast carcinoma	35	10	7	5
6	Breast carcinoma	31	8	6	4 ^a^
7	Mesothelioma	26	10	10	8
8	Hodgkin’s lymphoma	24	8	6	4
9	Hodgkin’s lymphoma	30	9 ^a^	25	25 ^b^
10	Breast carcinoma	32	6	7	7 ^b, c^
11	Breast carcinoma	30	10	10	5
12	Hodgkin’s lymphoma	25	13	4	3
13	Non-Hodgkin’s lymphoma	27	11	14	14 ^b, c^
14	Breast carcinoma (hormone receptor–positive)	33	11	13	7

^a^Stimulation started in the luteal phase.

^b^No ICSI; oocytes were cryopreserved in an unfertilized state.

^c^Received gonadotropin-releasing hormone agonists during cancer treatment.

### Ovarian stimulation

All of the patients had regular menstrual cycles before chemoradiotherapy. The phase of the menstrual cycle was evaluated using the onset of the last menstrual period, ultrasonography, and progesterone concentrations. Patients who were stimulated during the follicular phase received either a short “flare-up” protocol or a GnRH-antagonist protocol [6,7]. In the case of a single patient, stimulation was started in the luteal phase with a modified GnRH-antagonist protocol [8]. In one case, ovarian stimulation was carried out with letrozole in combination with a GnRH-antagonist protocol, due to estrogen receptor–positive breast cancer [9,10].

Follicular growth was monitored using vaginal ultrasound and measurement of 17β-estradiol (E_2_) levels. The gonadotropin dosage was adjusted according to the preantral follicle count and follicle growth. A single dose of recombinant human chorionic gonadotropin (hCG) was administered when the lead follicle had a mean diameter of 15–18 mm.

### Oocyte and ovarian tissue collection

Transvaginal oocyte retrieval was scheduled 34–36 h after hCG administration and was performed with the patient under general anesthesia. Immediately afterwards, ovarian tissue was extracted laparoscopically; the stimulated ovary was divided along the longitudinal midline with scissors, without the use of any diathermy. During this procedure, an effort was made to avoid coming too close to the ovarian mesentery, containing the ovarian vessels. In this way, the anti-mesenteric half of one ovary was separated and retrieved for cryopreservation, while the other half was left in situ. For hemostasis after this, circumscribed bleeding from the ovarian tissue was coagulated using bipolar diathermy. This was necessary at points along the surface of the ovary and on the ovarian septa between the former follicles.

The ovarian cortices were cryopreserved using a slow freezing protocol and an open freezing system [11]. Prior to freezing, a small biopsy of the ovarian tissue was examined histologically to assess follicle density and exclude involvement of the tissue by malignancy.

The mature (metaphase II, MII) oocytes were fertilized by intracytoplasmic sperm injection (ICSI) to avoid the risk of fertilization failure with in vitro fertilization (IVF) and cryopreserved at the pronuclear (PN) stage using a slow freezing protocol in accordance with the patient’s request and with German national law. If the patient did not have a partner or the patient requested it, all oocytes were cryopreserved in an unfertilized state using a slow freezing protocol.

## Results

All of the women underwent one IVF cycle. The median period of the hormonal stimulation cycle, between the start of hormonal stimulation and hCG administration, was 10 days (range 6–13 days). The average number of oocytes retrieved per patient was 10, and 67% of the oocytes were successfully fertilized using ICSI. In three cases, the oocytes were cryopreserved in an unfertilized state (mean number of unfertilized oocytes 15) (Table 1).

The transvaginal oocyte retrieval and extraction of the ovarian tissue immediately afterward were uneventful in all cases. No perioperative complications such as severe bleeding occurred. The fertility preservation procedures did not lead to postponement of the start of chemotherapy for any of the patients; however, one patient developed a mild ovarian hyperstimulation syndrome (OHSS).

**Figure 1:**
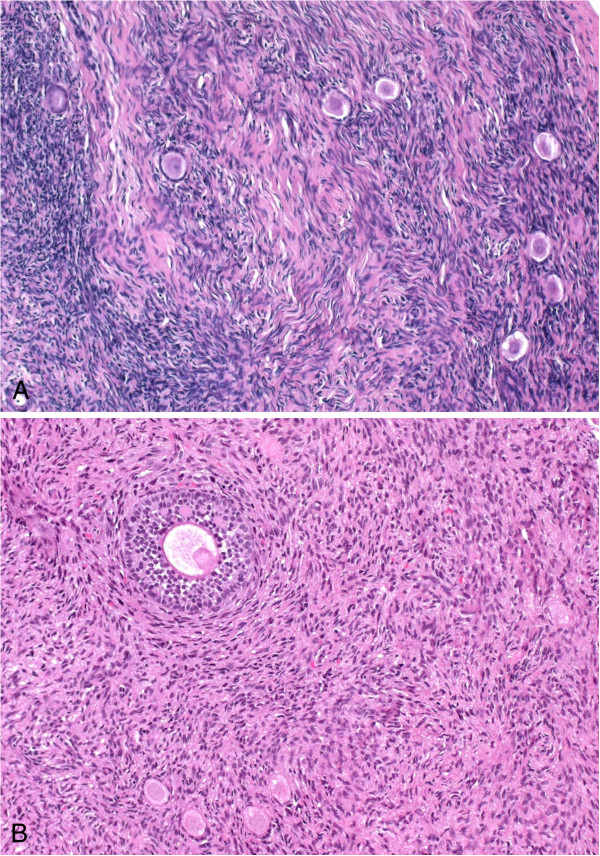
**Histological analysis of ovarian tissue extracted immediately after ovarian stimulation.** Ovarian tissue with (**A**) follicles and (**B**) one secondary follicle, stained with hematoxylin–eosin (original magnification: × 20).

The prefreezing histological sections from the human ovarian grafts had a normal histological appearance. The follicular count showed a normal age-related follicular distribution, with the vast majority of follicles being primordial. No carcinoma cells were seen in any of the hematoxylin–eosin-stained slides examined (Figure 1).

## Discussion

Ovarian stimulation, followed by intracytoplasmic sperm injection and cryopreservation of embryos, is currently the most successful procedure for fertility preservation in newly diagnosed cancer patients. Depending on the patient’s age, a survival rate of the embryos following thawing of 35–90%, an implantation rate of up to 30%, and a cumulative pregnancy rate of 30–40% can be achieved [12,13].

Freezing unfertilized oocytes is also a promising option for preserving fertility today. Oocyte banking does not require any partner or sperm donor and it may also accord better with various religious or ethical considerations than embryo freezing. With recent improvements in freeze–thaw protocols such as vitrification, promising results with more than 60% of mature oocytes surviving after thawing and subsequent fertilization have been reported — rates comparable with fresh oocytes [14,15].

For either of these methods to be successful, however, appropriate quantities of oocytes have to be obtained. In addition, because the time frame up to the initiation of chemotherapy and/or radiotherapy is limited, usually only one IVF cycle can be carried out, and the numbers of oocytes or embryos cryopreserved are consequently often not sufficient for several transfer attempts. For maximum effectiveness, combinations with other fertility preservation techniques therefore need to be considered.

Cryopreservation of ovarian tissue offers an effective combination. Cryopreservation of ovarian tissue before oncologic treatment has recently become one of the most promising techniques for preserving fertility. It allows storage of a large number of primordial and primary follicles. It can be carried out rapidly at any time in the menstrual cycle without delaying the oncological treatment and provides a unique option for preserving fertility in prepubertal or premenarchal female patients [16]. However, the method is surgically invasive and there is a potential risk that malignant cells in the frozen tissue may lead to recurrence of the primary disease after transplantation. For most conditions, however, the risk is low and is presumably related to the stage of disease at the time of ovarian tissue cryopreservation, although considerable caution is advisable with cryoconserved tissue from patients with leukemia, borderline ovarian tumor, or with a high risk of ovarian metastases (e.g., in adenocarcinoma of the cervix or stage III–IV breast cancer) [17]. A total of 20 live births have been reported to date after orthotopic transplantation of cryopreserved ovarian tissue [18-21]. Although cryopreservation of ovarian tissue is still considered experimental, the technique is now gaining worldwide acceptance.

In the cancer patients included in the present study, ovarian stimulation was carried out first, followed by laparoscopic collection of the ovarian tissue. Although it has been reported that ovarian tissue is of poor quality after ovarian stimulation [22], no data on this topic have so far been published. Histological examination of the ovarian tissue showed a normal age-related follicle distribution. No histological differences were found from ovarian tissue from patients who underwent ovarian tissue cryopreservation in our department without prior ovarian stimulation. Nor was any correlation noted between the numbers of oocytes retrieved and the follicle distribution in the ovarian tissue. In patients with fewer retrieved oocytes, the numbers of follicles were similar to those in patients with a high response to ovarian stimulation.

The ovarian response to stimulation is crucial for successful fertility preservation, and there has been concern regarding the ovarian response to ovarian stimulation in cancer patients. In the present study, different stimulation protocols were used due to the different starting days for stimulation. Adequate numbers of oocytes were retrieved within 2 weeks. The average number of oocytes retrieved per patient was 10, and 67% of the oocytes were successfully fertilized. This is in accordance with recent studies that have reported no significant changes in the ovarian reserve or response to gonadotropins in patients with various types of cancer [19,20]. However, other studies have reported a poorer ovarian response in cancer patients undergoing IVF treatment protocols [17,18]. The published data on this topic are still inconsistent.

The present group of patients included five women with breast cancer, one of whom had estrogen receptor–positive breast cancer. Concerns have been raised regarding the use of controlled ovarian stimulation in patients with hormone-dependent tumors, due to inadequate data on short-term increases in hormonal effects on the tumor. Moreover, as animal models suggest, estrogen may also play a role in stimulating the growth of estrogen receptor–negative breast cancers [23]. Conventional stimulation protocols with gonadotropins are therefore modified to include administration of the aromatase inhibitor letrozole [9,24] or the selective estrogen modulator tamoxifen [25]. These protocols have been used with success in reducing the estradiol excesses that are normally seen with conventional protocols, and short-term follow-up data for these protocols have not shown any detrimental effects on survival [10].

The risk of ovarian hyperstimulation syndrome (OHSS) is a known complication of controlled ovarian stimulation. One patient in the present study developed a mild OHSS, but the start of cancer treatment did not have to be postponed in any of the patients. The overall risk of severe OHSS is low, and in cancer patients it is also reduced, given that pregnancy will not occur; however, the risk should not be underestimated. Careful selection of the gonadotropin starting dosage, close monitoring, and step-down dosing are critical for avoiding complications. Triggering using a GnRH agonist alone or together with low-dose hCG might potentially further reduce the risk of hyperstimulation [26,27].

A potential side effect of the subsequent use of oocyte retrieval and ovarian tissue extraction may be bleeding in the residual ovarian tissue. Stimulated ovaries are more fragile than unstimulated ovarian tissue, which has a more compact structure. Stimulated ovaries have to be handled with greater care in comparison with unstimulated ovaries, to avoid injuries to the surface and to minimize possible tissue damage and bleeding. However, no side effects of this type were observed in any of the patients.

Several attempts have been made to improve the effectiveness of fertility preservation programs by combining different techniques. Removing ovarian tissue first and starting ovarian stimulation approximately 1–2 days later is an effective alternative approach. The partial removal of ovarian tissue does not substantially affect the average number or quality of oocytes retrieved after ovarian stimulation [22].

The combination of cryopreservation of ovarian tissue before chemotherapy and ovarian stimulation after the start of chemotherapy should no longer be carried out, as the efficacy of IVF is dramatically reduced even after one round of chemotherapy and high rates of malformation of offspring after treatment with alkylating agents have been demonstrated experimentally [28,29].

If there is no time for ovarian stimulation, cryopreservation of oocytes retrieved during dissection of resected ovarian tissue has also been reported as a potential strategy for preserving fertility in patients with cancer. The combination of in vitro maturation (IVM) with oocyte cryopreservation prevents any delay in cancer treatment and avoids the risks associated with high estradiol levels in hormone-sensitive tumors [30]. Although healthy infants have been born following IVM, implantation and pregnancy rates are generally lower than for IVF with mature oocytes [31,32].

Administration of GnRH-agonist analogs, in an attempt to reduce the gonadotoxic effects of chemotherapy by simulating a prepubertal hormonal milieu, is another fertility preservation method and should be combined with other fertility-protecting measures as well if possible. Although conclusive proof is still awaited, there is increasing evidence that GnRH agonists are effective in protecting the ovaries [33]. Administration of these agents may be considered on an individual basis, as the method is safe, noninvasive, and easy to administer.

## Conclusions

Fertility-preserving procedures should be offered to all patients facing fertility loss before cytotoxic treatment is administered. The decision as to which fertility preservation treatment is most suitable in the patient’s individual situation has to be made during a personal discussion with her and requires intensive interdisciplinary discussion, including oncologists, radiotherapists, and reproductive medicine specialists. A combination of fertility preservation techniques increases the efficacy of the procedure and gives young cancer patients the best chance for future fertility [5,34].

## Abbreviations

GnRH: Gonadotropin-releasing hormone;hCG: human chorionic gonadotropin;ICSI: Intracytoplasmic sperm injection;IVF: in vitro fertilization;IVM: in vitro maturation;OHSS: Ovarian hyperstimulation syndrome;PN: Pronuclear (stage)

## Competing interests

The authors hereby declare that they have no competing interests.

## Authors’ contributions

LL, AM, RD, and TH designed the study and analyzed the data. LL and RD wrote the manuscript. TH collected the data. AM, TH, and MWB carried out the operations. DW, LL, KUA, and IH carried out the histological work and analysis. MWB and RD supervised the study. All of the authors read and approved the final manuscript.
